# Realist evaluation of factors affecting the retention of the mental health workforce: building a realist programme theory

**DOI:** 10.1136/bmjopen-2025-102161

**Published:** 2025-08-31

**Authors:** Emily Wood, Sally Ohlsen, Jaqui Long, Elizabeth Lumley, Michaela Senek, Joe Hulin, Rachael Finn, Tony Ryan, Scott Weich

**Affiliations:** 1 SCHARR, The University of Sheffield, Sheffield, UK; 2 Sheffield University Management School, The University of Sheffield, Sheffield, England, UK; 3 School of Allied Health Professionals, Nursing, and Midwifery, The University of Sheffield, Sheffield, UK

**Keywords:** MENTAL HEALTH, Job Satisfaction, QUALITATIVE RESEARCH

## Abstract

**Abstract:**

**Objective:**

The shortage of mental healthcare workforce is a global problem. Mental health services have higher staff turnover and more vacancies than the health sector average. In this study, we developed, tested and refined an initial programme theory (IPT) to offer insight into mechanisms that affect staff retention in UK mental health provider organisations.

**Design:**

A realist evaluation to identify the contexts and mechanisms that impacted on workforce retention. We developed an IPT through a realist review and stakeholder engagement.

**Setting:**

We then tested the IPT through a realist evaluation in six case studies in selected National Health Service (NHS) mental health Trusts in England. Sites were selected to give variation in retention rates; quality ratings were awarded by the national healthcare regulator, staff satisfaction levels and geography.

**Participants:**

Realist interviews (199) were conducted with senior executives and registered mental health clinicians. Data were analysed using an iterative realist approach to analyse the links between the context, mechanism and outcomes and test and revise initial programme theories. The study has been reported in line with Realist And Meta-narrative Evidence Syntheses: Evolving Standards (RAMESES) II guidelines.

**Results:**

Data allowed us to refine the IPT. Workplace contexts influenced the key mechanisms of ‘perceived quality of care and the ability to make a difference’, ‘psychological safety’, ‘feeling valued’ and ‘feeling connected’, all of which influenced intention to leave.

**Conclusions:**

The final programme theory highlighting key contexts and mechanisms for improving retention among mental health staff in the NHS is presented. Organisational culture underlies all other contexts and mechanisms.

**STRENGTHS AND LIMITATIONS OF THIS STUDY**

- Use of realist methodology enabled development of a rigorously tested programme theory of the reasons for staff leaving, rather than simply identifying retention as a problem.
- A large sample size, drawn from a diverse range of settings and roles, increases confidence in the reliability of the findings.
- The sample was drawn from the current workforce, meaning the perspectives of those who had left the organisation are missing, although many participants reported changing roles or settings.
- Only registered staff working in English National Health Service Trusts were included, limiting wider applicability.
- No patients or service users were included, which may mean some factors impacting on retention have not been identified.

## Introduction

 Globally, across the healthcare sector, there are significant workforce shortages.^1^ The global mental health workforce faces similar challenges, with many healthcare professionals pursuing alternative employment opportunities.^2 3^ In England, where the National Health Service (NHS) is the primary provider of mental health services, there is a notable disparity in turnover between mental health services and the NHS overall. In 2021/22, 13% (17 000 staff) of all mental health staff left their positions compared with 11% across all NHS employers, resulting in an increase in vacant positions.^2 3^ The repercussions of staff shortages and high turnover extend beyond individual practitioners, affecting the remaining staff and the organisation as a whole in what has been labelled the ‘doom loop’,^4^ where increased workloads and poor staff morale add to the risk that others will leave. Recent staff surveys in England show that more than one third of mental health staff had experienced work-related stress significant enough to make them ill.^5^ High turnover also negatively affects the organisation as recruitment and training incurs significant costs.^6^ As many mental health interventions rely on trusting therapeutic relationships, high turnover of mental health staff also has an adverse impact on patient satisfaction, quality of care and outcomes.^7^ Given the wide-ranging impact of this turnover on staff, patients and healthcare organisations, it is important to understand the complexity of interweaving factors affecting retention of mental health clinicians, so that these can be addressed.^7^

Recent UK Government publications recognise workforce retention as a health policy priority.^8^,^11^ However, most previous studies have focused on retention in specific mental health professions such as mental health nurses,^7^ primary care-based mental health workers^12^ and psychiatrists^13^ rather than the entire mental health clinical workforce. Equally, the study on psychiatrists focused on presence or absence of burnout rather than its causes,^13^ although they did highlight the concern over growing workloads and the impact that had on quality of care. Retention is known to vary among settings, services and between professions,^14^ but the reasons for this variation have not been fully explored in order to understand what factors affect whom and in what circumstances. Despite this lack of understanding, many studies have focused on developing and testing generic staff interventions, which have often proven ineffective.^15 16^

This study adopts a realist methods approach to provide insight into the mechanisms that are triggered or inhibited in specific contexts to elucidate how certain outcomes (such as improvements in quality and safety, effective team working and staff satisfaction) are achieved. Realist evaluation provides a detailed and nuanced understanding of complex issues like staff retention by not only identifying the factors that influence mental health workforce retention but also examining the mechanisms through which these factors operate and the circumstances that shape their effectiveness.^17^

This study addresses the critical issue of clinical workforce retention by investigating the factors contributing to variation across multiple case studies in mental health settings. Recognising the complexity of healthcare organisations, a realist evaluation approach is particularly well-suited, as it accounts for the interplay between different organisational contexts and the mechanisms that influence outcomes. This approach enables the development of a nuanced programme theory that can inform future interventions and strategies.^18^

To our knowledge, this is the first study to adopt a realist methodology to explore how varying contexts activate specific mechanisms that impact clinical mental health staff retention. By building a programme theory, this study examines the interconnected contextual conditions, mechanisms and outcomes to provide valuable insights into the pathways and factors influencing workforce retention. These findings aim to inform actionable recommendations and guide future research and implementation strategies.

## Methods

### Design

This study followed the realist evaluation methodology offered by Pawson and Tilley^17^ and adapted for multiple organisational case studies,^19^ which allowed us to explore what it is about the context that affects whether mechanisms are activated to produce particular outcomes across different organisations.

Realist evaluation is an iterative, theory-driven approach that explores ‘what works, for whom, in what context, to what extent, how and why’.^17^ It acknowledges the complexity of organisations and systems, examining the interplay of micro-level and macro-level processes. Rather than focusing solely on inputs and outputs, it identifies underlying mechanisms (M)—the internal reactions and reasoning triggered by an intervention—that lead to specific outcomes (O) within particular contexts (C).^17^ These relationships, expressed as context-mechanism-outcome configurations (CMOCs), form the foundation of the initial programme theory (IPT), which is continuously developed, tested and refined to explain why interventions, such as workforce retention strategies, work in some contexts but not others.

The research was conducted in two distinct stages: the first stage involved a realist review of the literature alongside consultation with key stakeholders (reported elsewhere^20^) to develop an IPT (IPT); the second stage (reported here) involved semistructured, realist interviews with mental healthcare clinicians and senior managers across six mental health sites in England to refine and finalise the IPT.^21^ The method and results are described in line with Realist And Meta-narrative Evidence Syntheses: Evolving Standards (RAMESES) II guidelines on the reporting of realist evaluation research.^22^

### Realist review

**Figure 1:**
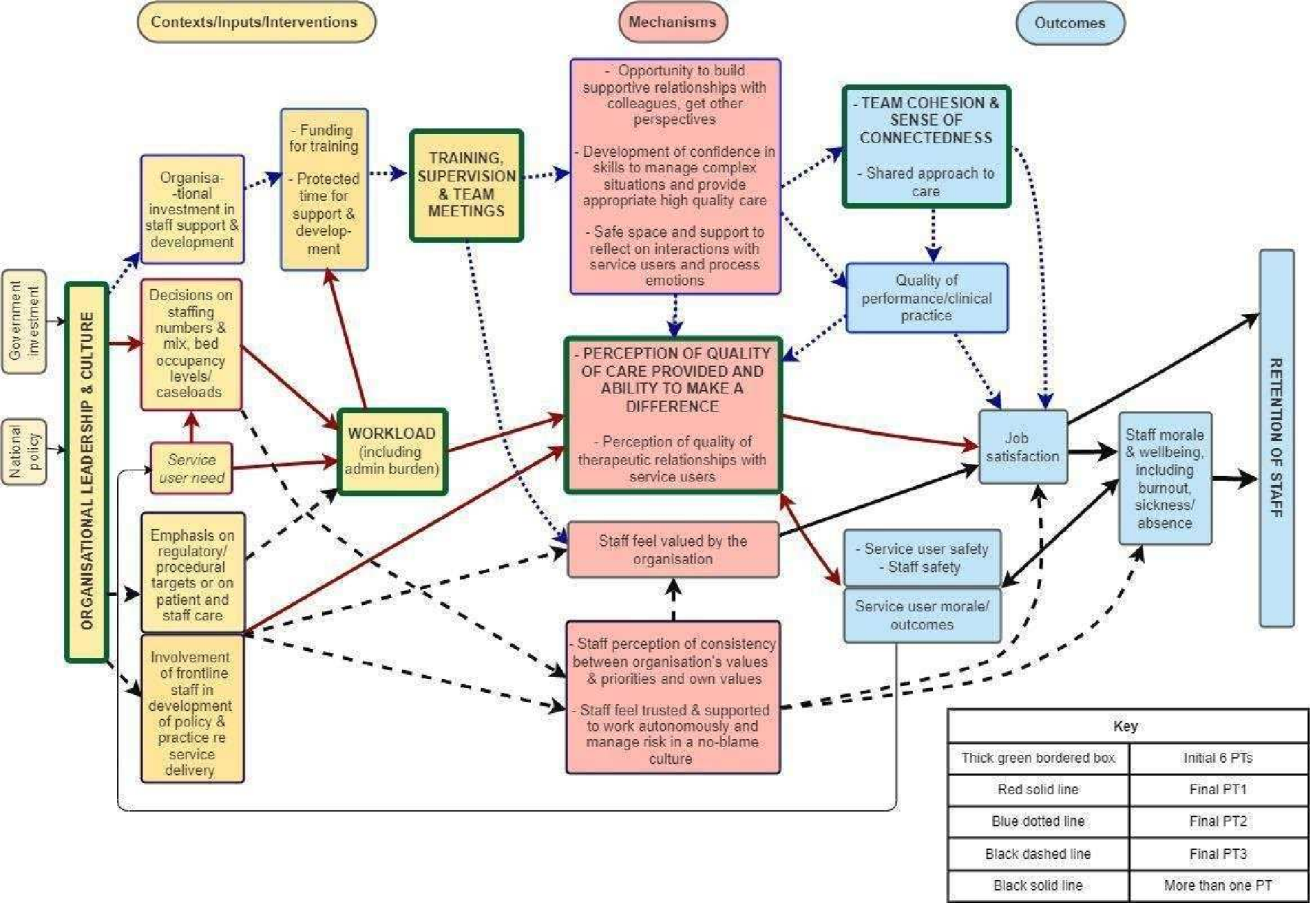
Initial programme theory (PT) of what contexts impact on retention in mental health settings from literature review (first published in the study by Long *et al*).^20^

The starting point for the evaluation was the development of the IPT through an iterative process of stakeholder engagement workshops and realist review of the published literature on the topic of mental health staff retention (full details in the study by Long *et al)*.^20^ The IPT went through a series of iterations due to the complexity and interwoven nature of factors which we identified as influencing healthcare workforce retention (figure 1). This was refined into three CMOCs (online supplemental table 2).

### Workforce interviews

In this step of the realist evaluation, we aimed to test, develop and further refine our IPT by exploring the mechanisms that affect clinical staff retention in mental health organisations in England. The scope (the retention of clinical mental health professionals in the English NHS) was predetermined, but the focus of the interviews was determined by the IPT, by identifying key mechanisms hypothesised to influence staff retention in mental health services. By testing and refining the IPT against real-world experiences, we could refine how different mechanisms were triggered in varying organisational contexts.

While many retention strategies are being tried in individual organisations, there is no standardised approach to this issue, and we therefore evaluated the ‘situation as usual’ and how that was affecting retention in the participating organisations.

Our objectives were:

- To interview frontline clinical staff about what influences their motivation to remain in post or to leave their role or the organisation.
- To interview senior staff about why they think people are leaving and organisational level strategies to improve retention and their perceived effectiveness.
- To modify the IPT to more clearly reflect the real-world experiences of our participants.

We used multisite case studies, undertaking realist interviews^23^ with senior management and frontline clinical staff (n=199) at each site to address the research question ‘In the context of mental health services, what factors influence staff retention, for whom, why and under what circumstances?’ The topic guide was structured to cover the key areas identified in the realist review but was sufficiently open to allow for the emergence of new themes.

Results from different sources and different organisations were triangulated using comparative methods to elucidate a theory and national set of recommendations relating to retention. This study was conducted in the NHS in England, a publicly funded national healthcare system. Most services and treatments are free at the point of need and provided by organisations called Trusts. Trusts typically provide mental health services for those who live in a defined geographical area. At the time we conducted this study, there were 54 mental health Trusts within England providing a range of services, generally delivered either in community or inpatient settings. Almost all services are staffed by multidisciplinary teams of registered and unregistered clinical staff, supported by a range of administrative services. This study focused on registered clinical staff (eg, physicians, nurses, occupational therapists, psychologists and social workers) and senior managers (who may or may not be registered clinicians).

### Site selection

Six English mental health Trusts were purposefully selected by the research team to be case study sites. Sites were selected to ensure variation in the following criteria:

- Retention rates of clinical staff (based on the annual stability index).
- Job Satisfaction score on NHS staff survey.
- Care Quality Commission (CQC) ratings (national inspection body for healthcare providers).
- Geographical location, encompassing urban, rural and mixed settings across England.

To protect anonymity, the sites are identified by letters A–F.

Three Trusts were in the South of England, two in the North and one in the Midlands. Two served urban populations, the rest served a mixture of rural and urban. Stability index in the year prior to data collection ranged from 0.795 to 0.885. Staff satisfaction ranged from two in the bottom five ratings for all English Trusts to two in the top 10 for staff satisfaction.

### Sample and recruitment

At each site, 23–45 frontline registered clinicians and senior managers (directors or their delegates or people with specific responsibilities around retention and workforce) were interviewed to discuss what they thought were the key factors motivating staff either to leave or to remain in post. Data collection took place between March and November 2021. As this coincided with travel restrictions in the UK due to the COVID-19 pandemic, all communication was carried out using remote methods. Recruitment emails and information sheets were sent out via research and development (R&D) and communications teams. In most cases, these were sent to all clinical staff as well as targeted emails to senior managers and staff networks. Some organisations did not have all-staff mailing lists, so they used multiple professional lists to reach as many staff as possible. Potential research participants responded directly to the research team by completing an online consent form. Organisations were not informed who responded to the invitations. Purposeful sampling was employed to try and address under-recruiting in key groups. For example, where we did not recruit many participants from inpatient services in response to the initial recruitment email, R&D staff approached ward staff directly or used specific mailing lists to target them. These approaches were bespoke to the situation in each organisation.

All registered staff who were employed by the organisation were eligible regardless of job title.

### Data collection

Data were collected via semistructured interviews with participants by telephone or online using video conferencing software. Interviews were based on a topic guide which allowed for exploration of the IPT as well as novel themes. Pilot interviews were used to test the topic guide on clinical/academic colleagues, which led to some minor changes. The topic guide was subsequently adapted during data collection to further explore new issues and emerging categories. The interviews lasted between 40 min and 80 min. All interviews were audio-recorded and transcribed. At the start of all interviews, participants were reminded that their participation in the study was voluntary, they could withdraw at any time and the research team would maintain their anonymity. No participants decided to withdraw once they had completed the interview, but not all staff who consented were interviewed due to the research team not being able to make follow-up contact or withdrawal prior to interview due to workload commitments and capacity. Interviewers (five researchers, EW, SO, JL, EL, MS) met weekly to review the topic guide and emerging themes in line with constant comparative methodology.^24^ Many of the interviews were highly emotive and the weekly interview meetings were also used for debriefing and peer support for the research team.

### Data analysis and synthesis

A deductive, iterative approach to analysis was taken, with multiple cycles to test, refine and develop the IPT. This began on the micro level with analysis of the individual interviews, then moved to the meso level exploring patterns (CMOs) and themes within each case study and finally to the macro looking at the themes across the study sites. Qualitative coding software (Quirkos) was used to facilitate data management and analysis. The analysis was undertaken by four members of the research team (EW, SO, JL, EL). Initially, all four researchers coded the same selected transcripts to develop consensus around use of the coding framework. They then coded independently but regularly met to discuss any uncertainties regarding coding and to reach consensus.

The initial coding of individual interviews across all case studies was based on codes from the IPT,^18 20^ and emerging additional codes were incorporated after team discussions. In a second round of analysis, emerging themes and patterns across the case study sites were explored. Developing themes were used as a basis to dispute the IPT and refine the model.

While there was a patterning of experience which was unique to each case study site, our second stage of analysis using cross-case comparisons to determine how the same mechanism (such as ‘making a difference’) or submechanism (such as ‘feeling valued’ or ‘feeling trusted’) operated in different contexts (eg, workload, organisational culture, availability of supervision) and produced different (or similar) outcomes (ultimately retention rate, but also job satisfaction and staff well-being). This stage aimed to explore inferences about the generative causality of different contexts. This was done in iterative cycles using analysis research team meetings to test and retest similarities and differences between context and mechanisms and how and why they might lead to a range of outcomes. This process was the basis for the revision of the programme theory.

### Patient involvement

Patient and public panels were involved in the design and conduct of this study. For details, please see the study by Long *et al.*^20^

## Results

Across the six organisations, 199 semistructured interviews were conducted. Those interviewed included 29 senior staff (such as chief operating officer, chief strategy officer, chief nursing officer, chief medical officer, chief people officer, two heads of human resources and deputies, equality, diversity and inclusion leads and workforce leads), 94 nurses, 28 psychiatrists, 26 occupational therapists and 22 psychologists (see participant characteristics table in the online supplemental appendix for more details).

### Modification of the IPT

The IPT (figure 1) demonstrated a complex interplay between multiple CMOs affecting staff retention.^20^ Although three key CMOCs were initially identified by the review (online supplemental materials), the system’s interdependent nature made it difficult to extract actionable policies and procedures to improve retention. The interview data provided crucial insights that allowed for a refinement of key pathways and the development of practical recommendations.

The refined CMO configurations derived from the interview data largely aligned with the IPT but differed in the details. Most notably, the interviews underscored the pivotal role of teams and team leaders in shaping retention. Participants described how a cohesive team could counteract the negative effects of a poor organisational culture, while a dysfunctional team could undermine even a well-functioning Trust.

As with the IPT, CMOCs were also not linear progressions as is often seen with traditional representations in the realist literature.^17^ For example, team cohesion could be an outcome (O), as seen in the IPT, but it could also be a context (C) that influences the way people feel supported (or not) in the workplace (M). Equally, although ‘perceived lower quality of care’ could be an outcome, in terms of staff retention, it is a mechanism that drives staff dissatisfaction.

### Key mechanisms and their influences on retention

#### Perceived quality of care

Confirming and reinforcing the IPT, participants constantly highlighted the link between workload and their ability to deliver high quality care and remained a key influence on job satisfaction and retention. Across inpatient and community settings, factors such as patient acuity, staffing levels, caseload size and administrative burden (C) interacted to shape staff perceptions of care quality (M).

*It’s crisis management (C), there is no actual recovery focus at all* (M – perceived quality of care). (Trust D Participant 14)

Participants discussed a variety of aspects of their workload (C) including patient acuity, staffing levels, caseloads and team compositions (experience/profession ratios/agency workers and how that affects delegation) alongside an increasing paperwork burden. Across all Trusts, frontline staff reflected on an increase in their workload, particularly over recent years (C). They reported how reductions in the number of inpatient beds (C) and an increase in patient acuity (C) were interacting and impacting on whether staff felt they could deliver good quality of care (M) in both community and inpatient settings. There were concerns that patient acuity was rarely considered in workload/staffing plans. Many staff reported that where there was no safe staffing level on wards or capped caseloads in the community, their workload became unmanageable, and they were unable to provide the quality of care they wanted to and were reduced to ‘firefighting’ and ‘risk management’.

*Staff saying that they don’t have the time to provide the quality and the level of care that they want (M)….it’s a mini time bomb… the frustration can only be carried for so long before staff vote with their feet (O*). (Trust A Participant 06)

Moral injury occurs when a staff member wants to act in a certain way but feels they are prevented in doing so by the organisation.^25^ The inability to deliver therapeutic care (C) was viewed as a moral injury (M) by all professions and contributed significantly to job dissatisfaction (O) and lack of desire to remain in post (O). This issue was particularly pronounced in Trusts with lower CQC ratings, where the combination of high patient acuity and inadequate staffing exacerbated dissatisfaction and intent to leave.

#### Psychological safety

Psychological safety in healthcare is defined as ‘an environment where everyone feels included, safe to ask questions and able to work without fear of retribution or retaliation’.^26^ Although psychological safety was implicit in some elements of the initial development of the IPT, it was not explicitly stated within the theory. However, it was clear in the interviews that this was a major mechanism influencing staff satisfaction, well-being and retention (O). One way this was evident was in perceptions of a ‘blame culture’: while senior managers tended to assert that the organisation was supportive, many frontline staff described expecting to be blamed if anything went wrong, for example, a serious incident. This perception of blame culture (C) led to feelings of stress, a reluctance to take positive risks and to a defensive approach to practice (M). This, in turn, led to reduced job satisfaction and a belief among frontline staff that they are not able to do the best for their patients (O), linking back to the quality-of-care theme.

*I think the difficulty is people go to A&E and they'll say I'm going to kill myself. So historically five, six, 8 years ago we would've done a really good risk assessment, we would've used some therapeutic risk taking. We don't do that anymore because we're scared of that person dying, having to go to coroner’s court. There’s a real fear*. (Trust D Participant 1)

Staff talked about feeling ‘traumatised’ by the way that incidents had been handled, and this was specifically linked by some participants to a desire to leave. Staff also reported a range of physical health impacts as a direct result of work. Inpatient staff disclosed experiencing or witnessing a range of physical injuries sustained in assaults from patients, which ranged from being scratched to being knocked unconscious. These incidents were compounded on an emotional level when there was a lack of support, leaving staff feeling undervalued and disposable.

*I’m just going to go home then, I’m going to go home because no one cares that I’ve been really severely assaulted, no one’s even asked if I’m ok*. (Trust C Participant 56)

Part of the refinement on the IPT which came directly from the realist interviews is the focus on inclusion, anti-racism and tackling of bullying within organisations (both in actions and policies) and how this can influence not only staff’s psychological safety (M) but also their sense of being valued (M) and connectedness to a local team or healthcare organisation (M). For example, while each Trust reported a visible range of staff support and development and training opportunities (C), the degree to which these were offered to staff in a fair and inclusive way was often more important than their existence (M). Staff reported in many Trusts the lack of equitable distribution of resources and opportunities. They also highlighted a lack of transparency on decisions on which staff were put forward for promotion or were able to access training and development opportunities, particularly where this involved funding (C). This led to staff feeling unfairly treated and demoralised (M).

*Recently there is a flurry of people who have gone from our Trust to a local Trust (O), because they felt that … as a person from a different ethnic background (C), you won't be able to progress (M*). (Trust D participant 25)

While issues relating to this theme were discussed across all Trusts, there was a notable variation in how central it was to participants’ concerns which appeared to relate to the Trust’s CQC rating. Training and development and their impact on retention had more prominence in interviews in Trusts with higher ratings. In these Trusts, staff appeared to have the energy and time to focus on these wider issues, whereas in underperforming Trusts, staff (and the organisation) were more concerned about core issues of workload and quality of care and seemed to have no capacity to think about their own development.

### The impact of organisational culture on feeling valued

Feeling valued was seen as a key mechanism which influenced staff’s job satisfaction and morale and retention. Underpinning many of the issues highlighted by participants was their experiences and perceptions of the organisation’s culture (C), and its direct influence on how it left staff feeling valued or undervalued. Aspects of this were identified within the review, but the interview data extended this substantially and emphasised its significance. Participants identified the importance of an organisational culture that embedded listening to, engaging with and responding to clinical staff at the core of its policies and practice, demonstrating an open and learning culture (C) with staff being valued as a central cog. Key issues raised included having trust in the leadership that staff were valued (M), leadership valuing the variety of staff opinions (M) and the organisation valuing transparent and accountable culture across all staffing groups (M).

Several participants described structural reorganisations within their Trust (C) which had left a legacy of mistrust (C). Participants pointed to the pain and feelings of destabilisation that this caused (C). When staff feel undervalued and unappreciated (M), their morale decreases (O), and they may consider alternative employment (O). It also makes future restructuring extremely stressful (O).

*We’ve had difficult changes, but we had a reconfiguration which was handled really badly and that’s left some people feeling very bitter and risk wasn’t managed well during that process…* (Trust B Participant 07)

More generally, where Trusts employed a top-down approach to embedding policies and practices (C), staff lacked confidence in the Trust and were cynical about change (C). If organisations were perceived to prioritise financial or numerical targets (C) rather than staff and service users, participants felt little sense of belonging (M) and loyalty (M) and reported increased dissatisfaction in their jobs (O). In contrast, where meaningful consultation and engagement occurred (C), there was more reported engagement and job satisfaction (O). The notion that individuals can influence change was key (M). Many highlighted the importance of the organisation’s leadership in engaging people with changes and responding to their ideas through meaningful consultation (C) as key to successful retention (O). In contrast, where staff reported how senior staff listened but did not respond and let them know what action had been taken (C), this left them frustrated and feeling undervalued (M).

*They’re massively changing some of the structures, and teams have been informed about it when the decision’s been made and it’s happening rather than a consultation of, this is what we want to do, this is why we have to do it, what do you think, which is what would be preferable*. (Trust C Participant 48)

### Feeling connected: the key role of line managers and team leaders

Feeling connected and the sense of belonging came across strongly in the review as a mechanism influencing retention, but the pivotal role of middle level managers such as team leads and ward managers in staff retention was not identified from the review and IPT but was very evident within the interviews. Team leadership was linked strongly to having a sense of belonging (M) and staff described how team leaders had a key role in anchoring staff in both their professions and posts within an organisation (M). Staff reported actively seeking roles in stable teams with team leaders who had good track records (C); this information often spread via word of mouth. Conversely, other teams or environments where staff reported a lack of connection with team leadership (M) were avoided, leading to under-staffing (O) due to the lack of team leadership (C) and instability within the team (C).

*The single most critical point though is the quality of the manager (C). One of my favourite sayings is… you join an organisation, you leave your manager (O*). (Trust A Participant 06)

A good manager (C) was seen as someone who is strongly supportive (M) and available and accessible to all members of the team (M). Managers who had a clinical background (C) were generally better respected and valued than those who did not (M). While some people have natural leadership skills, the need for robust and consistent management training was regularly discussed.

However, several staff at all levels reported that middle managers were often themselves unsupported, pressured, asked to do the impossible and even bullied by the higher-level leadership (C). This led to high turnover in these positions (M) and instability and lack of support within affected teams (O, C).

### Refining the programme theory

**Figure 2:**
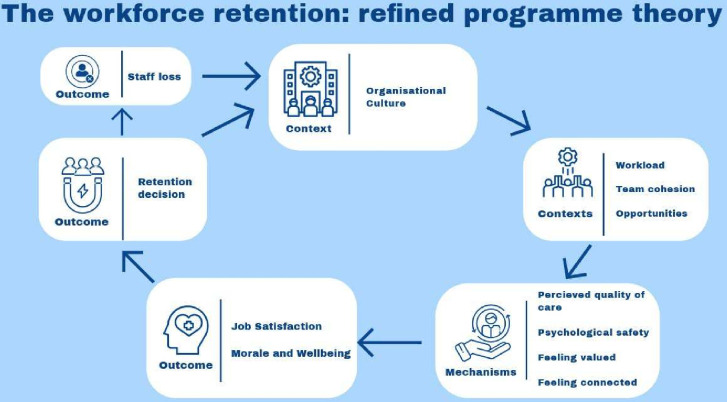
A depiction of the final programme theory.

While very little data from the interviews directly contradicted the IPT, the analysis led to a change in emphasis in some sections and the identification of new mechanisms. This suggests that previous research has failed to identify some ways in which retention is enhanced or undermined among mental health staff, including the influential roles played by team leaders and the need for psychological safety. Psychological safety is a mechanism that was not identified in the initial literature review or development of the IPT but was something that participants regularly mentioned and has therefore been integrated into the refined model. This was strongly linked to the support provided by leaders and teams as well as to workload levels. The model has been reworked (figure 2) to reflect these changes and the non-linear nature of the relationships.

In this non-linear model, organisational culture (C) unpins the other contexts within the organisation and determines if workloads are manageable, cohesiveness of teams and the opportunities and development that are available to staff (C). These secondary contexts impact on the daily working lives of staff and affect whether they feel psychologically safe, connected and valued by the organisation and their colleagues, as well as if they judge themselves as able to give good quality care (M). These mechanisms are instrumental in the way a person feels about their role and their organisation and lead to outcomes such as job satisfaction and morale and well-being (O). Job satisfaction, morale and well-being are the decisive factors in determining intent to leave (O). Some staff will leave, and some staff will stay; both will then impact on organisational culture. Is this a place where happy, satisfied staff stay or overworked staff leave? Thus, triggering the cycle to turn again.

## Discussion

### Summary of principal findings

The interview data reinforced the crucial role of organisational culture as a factor affecting retention of mental health staff and the importance of workload and the perception of the quality of care the patients receive, both factors highlighted in the original IPT. The IPT was developed and refined; particularly highlighting the importance of psychological safety for frontline staff, the impact of the line manager and the perception of fairness and justice within the organisation.

### Comparison with existing literature

The recent NHS long term workforce plan^10^ recognises the importance of retaining staff. Many of the issues raised by our participants, such as the importance of well-led teams, organisational culture and reducing racism and bullying, are acknowledged, but there are, as yet, few plans to address these.^27^ The plan is welcome, but more robust recommendations are required, particularly in specialist sectors, as well as building on lessons learnt about how to shape and build cultures, which is notoriously complex, involving aligned structures, systems and processes versus narrow focus on ‘beliefs and attitudes’.^27^ The report also omits consideration of the causes of low retention, such as excessive workload and low psychological safety.

Understaffing, and therefore higher workload for the remaining staff, is a self-perpetuating problem and one that, if allowed to continue, adversely affects patient care.^28^ Participants told us that being overworked (C) and therefore feeling unable to provide as good quality care as you aspire to (M) leads to low morale (O) and eventually intention to leave (O). Safe staffing numbers in mental health settings would then be positive for staff and service users. However, the National Institute for Health and Clinical Excellence safe staffing guidelines for mental health were never published.^29^,^31^

20 years ago, Kleinman identified leadership as a key strategy for nurse retention,^32^ highlighting how leadership training and shared leadership models could be beneficial. Our findings support the need for high-quality leadership training at all levels of management and leadership in healthcare providers. This is important because leaders prepare and support frontline staff to work in challenging circumstances. This has been highlighted in specific environments, such as high secure facilities,^33^ but our findings would argue that this is important regardless of the setting. All mental health staff need psychologically safe and supported environments to work in.

Many of our participants did not feel psychologically safe. Many described a ‘blame culture’ which left them feeling defensive. This, in turn, led to moral injury. Moral injury was linked to low morale and intention to leave.^34 35^ Reports of blame culture were usually in direct opposition to senior staff in the same organisations who insisted that there was no blame culture.^36 37^ This highlights the importance of the final point in NHS Employers’ Top Tips for Psychological Safety, ‘Culture is not what is written in our policies, values, brand. It’s the everyday behaviours… from senior leadership down to all members of the support staff’.^26^

### Policy implications

There are implications for national level bodies, such as Government and NHS England, as well as organisational level recommendations for individual employers. The realist model shows that retention cannot be effectively addressed by focussing only on the end of the process. Organisational leadership needs to look at their contexts and the mechanisms activated in their organisations. Employers should focus on staff well-being rather than consider it a ‘nice to have’. Staff join the health service because they want to help people. They just need to be provided with a work environment conducive to making a difference, but our research has shown that many aspects of the organisational context undermine their capacity to do so. Change happens in health services all the time, however employers need to ensure it happens in collaboration with staff rather than being imposed on them. This can be done with meaningful consultation and two-way communication.^38^

Compulsory leadership and management training for all staff in any leadership or management role will help to improve management in services. Compassionate leadership training can reduce stress and improve staff engagement.^39^ This should include ongoing support and development, as our results showed that middle level managers are themselves squeezed from above and below. Training, development and career progression opportunities should be available to all staff through open, fair and accessible means. This includes considering the geographical location of training, ensuring staff can leave their workplace to attend without causing a staffing issue and that allocation of places is fair and equitable.

### Implications for future research

This study investigated the situation in mental health services during a time of significant pandemic restrictions and sickness, but participants did not in general consider that this created new problems; rather, it highlighted and exacerbated existing ones. However, the full after-effects of the pandemic will not have been evident at that time, and further research is needed to follow-up these questions.

This study investigated the situation as usual rather than the effects of a specific intervention. Future intervention studies to investigate retention strategies in health service organisations will be needed to understand further the nature of the interacting CMOs, particularly in relation to the impact of policy reconfiguration and reorganisation of mental health services.

### Strengths and limitations

By testing programme theories derived from existing literature in step one, the research aims to uncover valuable insights that can inform strategies to improve retention across different contexts. The lessons learnt from this investigation can contribute to a more nuanced understanding of the factors affecting frontline retention, with views spanning from frontline workers to senior executives and making meaningful comparisons between different mental health organisations and services.

The data were collected from NHS sites in England, and while healthcare systems vary globally, the fundamental challenges in managing complex emotional and mental health disorders are widespread. Further research is needed to explore these findings in different cultural and healthcare contexts. This is one of the largest qualitative studies ever carried out on healthcare staff and the large number of interviews across professions, settings and services gives us confidence that at least some aspects of our findings in relation to workforce retention are more widely transferable.

The study represents a snapshot of staff experiences up to December 2021; all data were collected during a period of COVID-19 restrictions. This may have impacted the data collected; the full impact of the pandemic on mental health Trusts remains an ongoing process. We adapted our topic guide to include COVID-19’s impact on retention.

This study did not interview service user representatives and does not therefore capture their perspective on workforce retention or its impact on quality of care, although mental health service user groups were consulted during the development of this research project, particularly the formulation of the IPT. However, the main aim was to discover staff perspectives and priorities, and this was achieved.

As we only included registered staff employed by the NHS organisation, we did miss the viewpoints of non-registered staff such as healthcare assistants and registered staff like social workers who are employed by the local authority rather than the NHS.

A key strength is the size of the study: our sample of 199 interviews is very large for qualitative research. This was necessary to ensure we had reached saturation across a wide range of professions and six sites. However, we did not achieve as much representativeness across all ethnic groups as we had hoped, and our remote data collection methods may have led to some exclusion of those who were less digitally confident and connected. Some perspectives may therefore be missing from this data.

## Conclusions

Maintaining the healthcare workforce represented a huge challenge for mental health providers; retention of the current workforce is only one strategy which healthcare policy must consider. The mental health workforce has been severely overstretched with an unmanageable workload, with a consequence of a significant negative impact on the well-being of staff, particularly those in frontline clinical roles. Staff in many sites reported an organisational culture where they did not feel supported, protected or valued and described a blame culture with a lack of psychological safety.

Key factors affecting healthcare staff retention are workload, organisational culture and psychological safety. Where an organisation limits staff caseloads (C), workloads remain manageable (C); this leads to staff feeling that they can do their job well (M), and this leads to improved job satisfaction (O), leading to improved retention (O). Equally, where staff feel supported and protected by their organisation (C), they feel able to take positive risks for the benefit of their patient’s treatment (M), leading to improved job satisfaction (O), leading to improved retention (O). The current government and policy priorities need to account for the need for creating these conditions if we are to address the chronic workforce challenges in mental health services and the wider NHS.

## Supplementary material

10.1136/bmjopen-2025-102161 online supplemental file 1

10.1136/bmjopen-2025-102161 online supplemental file 2

## Data Availability

No data are available.
