# Supplementary material for: Realist evaluation of factors affecting the retention of the mental health workforce: building a realist programme theory
Source: BMJ Open. 2025 Aug 31;15(8):e102161. doi: 10.1136/bmjopen-2025-102161 (PMC12406895; doi:10.1136/bmjopen-2025-102161)
Supplement: online supplemental file 1 [file bmjopen-15-8-s001.docx]

**Table A1 Participant characteristics table**

|  | | A | B | C | D | E | F | **Total Number Interviewed (%)** |
| --- | --- | --- | --- | --- | --- | --- | --- | --- |
| Total number interviewed | | 36 | 31 | 45 | 23 | 36 | 28 | **199** |
| Setting | Senior staff | 5 (14) | 4 (13) | 6 (13) | 4(17) | 4 (11) | 6 (21) | **29 (14)** |
|  | Clinical Community | 19 (53) | 17 (55) | 25 (56) | 8 (35) | 20 (56) | 14(50) | **103 (52)** |
|  | Clinical Inpatient | 10 (28) | 9 (29) | 14 (31) | 4 (17) | 9 (25) | 5(18) | **51 (26)** |
|  | Clinical Both | 2 (5) | 1 (3) | - | 7(30) | 3 (8) | 3(11) | **16 (8)** |
| Profession | Nurse | 17(47) | 16 (52) | 22 (49) | 11 (48) | 13(36) | 16 (57) | **95 (48)** |
|  | OT | 6 (17) | 5 (16) | 6 (13) | 2(8) | 8 (22) | 1(3.5) | **28 (14)** |
|  | Psychologist / Psychotherapist | 5 (14) | 2 (6) | 8 (18) | 1(4) | 3 (8) | 5(18) | **24 (12)** |
|  | Psychiatrist | 4 (11) | 4 (13) | 6 (18) | 3 (13) | 10(27) | 3(11) | **30 (15)** |
|  | Social Worker | 1 (3) | 2 (6) | 1 (2) | 3(13) | - | 2 (7) | **9 (5)** |
|  | Other | 3 (8) | 2 (6) | 2 (4) | 3(13) | 2 (6) | 1 (3.5) | **13 (7)** |
| Gender | Male | 10 (28) | 10 (32) | 15 (33) | 5(22) | 17(47) | 13(46) | **70 (35)** |
|  | Female | 26 (72) | 21 (68) | 29 (64) | 17 (74) | 19(53) | 15(54) | **127(64)** |
|  | Other/did not disclose | - | - | 1 (2) | 1(4) | - | - | **2(1)** |
| Ethnicity | White British | 25 (69) | 27 (87) | 42 (93) | 17 (74) | 29(81) | 15(53) | **155 (78)** |
|  | White Other | 5 (14) | 2 (6) | 2 (4) | - | 5(14) | 8(29) | **21(10)** |
|  | Black British | - | - |  | 1(4) | - | 1(4) | **2(1)** |
|  | Black Other | - | 2 (6) |  | 1(4) | - | 2 (7) | **6 (3)** |
|  | Mixed/Other | 2 (5.5) | - |  | 1(4) | - | - | **4 (2)** |
|  | British Asian | 2 (5.5) | - |  | - | 2(5) | - | **4 (2)** |
|  | Asian Other | 1 (3) | - | 1 (2) | 2(8) | - | 2 (7) | **5 (3)** |
|  | Did not disclose | 1 (3) | - |  | 1(4) | - | - | **2 (1)** |
| Length of Service within organisation  *Not full sample/  missing data (n=195) | Average length of service with in organisation | 9yrs | 11yrs | 11 yrs | 10yrs | 13yrs | 10yrs | **11yrs** |
|  | Less than 5 years | 13 (38)* | 10(32)* | 10 (22) | 6 (26) | 11(30) | 11(39) | **61 (31)*** |
|  | 5 years or more | 20 (59)* | 20(65)* | 35 (78) | 17(74) | 25(70) | 17(61) | **134 (69)*** |
| Length of Service in Post  *Not full sample/ missing data (N=195) | Average length in post (average) | 4yrs | 4yrs | 4 yrs | 4yrs | 6 | 5yrs | **4 yrs** |
|  | Less than 5 years | 26 (76)* | 23(74)* | 28 (62) | 16(69) | 24(67) | 20(71) | **137 (70)** |
|  | 5 years or more | 7 (21)* | 7(23)* | 17 (38) | 7(31) | 12(33) | 8(29) | **58 (30)** |
