# Supplementary material for: Realist evaluation of factors affecting the retention of the mental health workforce: building a realist programme theory
Source: BMJ Open. 2025 Aug 31;15(8):e102161. doi: 10.1136/bmjopen-2025-102161 (PMC12406895; doi:10.1136/bmjopen-2025-102161)
Supplement: online supplemental file 2 [file bmjopen-15-8-s002.docx]

Supplementary file

Table 2: Initial programme theory generated from the realist review, previously reported in Long et al 2023

| Brief title | CMOCs |
| --- | --- |
| Interconnectedness of workload and perceived quality of care | If the organisation’s leadership prioritises safe staffing levels to meet service user acuity and numbers (including a suitable balance of permanent and temporary staff on wards, capped caseloads in the community, and an appropriate mix of staff professions and experience) (context). THEN clinical staff perceive they have a manageable workload to enable them to deliver high-quality care, build therapeutic relationships and make a difference to service users (mechanisms). This LEADS TO increased job satisfaction, improved safety, and morale (for staff and service users); reduced stress and burnout and increased staff retention (outcomes). |
| Investment in staff support and development | IF the organisation’s leadership invests in the support and development of clinical staff (including protected time for quality supervision and regular supportive team meetings; and time and funding to undertake professional development) (context). THEN staff feel confident that they have the appropriate skills to provide high-quality care; have appropriate support to reflect on practice and process emotions; and believe they are valued members of the team and organisation (mechanisms). This LEADS TO improved clinical practice, better team relationships and a collaborative approach to service users’ care; increased job satisfaction, improved morale; reduced stress and burnout and increased staff retention (outcomes). |
| Involvement of staff and service users in policies and practice | IF the organisation’s leadership develops policies and practices which are informed by and involve clinical staff and service users in decisions about the delivery of services and prioritise targets relating to service user care and staff well-being (context). THEN staff feel listened to and valued, and perceive that the organisation’s values align with their own (ie, focusing on clinical need rather than targets and financial considerations) (mechanisms). This LEADS TO increased job satisfaction, improved morale; reduced stress and burnout and increased staff retention (outcomes). |
