# Supplementary material for: Realist evaluation of factors affecting the retention of the mental health workforce: building a realist programme theory
Source: BMJ Open. 2025 Aug 31;15(8):e102161. doi: 10.1136/bmjopen-2025-102161 (PMC12406895; doi:10.1136/bmjopen-2025-102161)
Supplement: Supplementary file 3 [file bmjopen-2025-102161.reviewer_comments.pdf]

PEER REVIEW HISTORY

BMJ Open publishes all reviews undertaken for accepted manuscripts. Reviewers are asked to complete a checklist review form and are provided with free text boxes to elaborate on their assessment. These free text comments are reproduced below.

ARTICLE DETAILS

Title (Provisional)

A realist evaluation of factors affecting the retention of the mental health workforce: building a realist programme theory.

Authors

Wood, Emily; Ohlsen, Sally; Long, Jaqui; Lumley, Elizabeth; Senek, Michaela; Hulin, Joe; Finn, Rachael; Ryan, T; Weich, Scott

---

VERSION 1 - REVIEW

**Reviewer**	**1**
**Name**	**Scanlan, Justin**
**Affiliation**	**The University of Sydney, Faculty of Health Sciences**
**Date**	**13-Apr-2025**
**COI**	**None**

Thank you for the opportunity to review this manuscript. The manuscript is well written and addresses an important issue for the mental health workforce.

I have only a few comments for consideration.

Line 124: the Pawson and Tilly citation needs to be corrected to be in line with the numbered citation system.

Throughout the manuscript "trust" is used to describe the organsiations.This can lead to challenges in readability at times. I would suggest capitalising as "Trust" to improve readability.

Line 405-406: "where staff reported how senior staff listened but did not respond and feedback" - I think that there is a word missing here... "where staff reported how senior staff listened but did not respond to feedback".

Line 513-515: "Compulsory leadership and management training for all staff in any leadership or management role will help to improve management in services and make it more supportive and compassionate (39)." - I think that this statement is a bit too definitive. While management training may be helpful, I don't think the evidence supports that it will

always create more supportive and compassionate workplaces. I would suggest revising this to be a more speculative suggestion.

Line 612 - this statement refers to "survey respondents" which doesn't seem to be aligned to the participants in this study.

**Reviewer**	**2**
**Name**	**Hancock, Jason**
**Affiliation**	**University of Exeter Medical School**
**Date**	**11-Aug-2025**
**COI**	**None**

Thank you for asking me to review this paper which addresses an incredibly important and timely issue. The paper is well written and easy to follow. The methodology used is well placed to answer the research questions identified and is clearly reported.

I have included some comments below relating to the reporting of some of the results but these are minor suggestions and do not detract from the quality of the work.

Choice of population and reporting:

I note in the introduction that this paper critiques existing research for only focussing on MH nurses, primary care based workers and psychiatrists when considering the factors that impact retention. The authors suggest that more evidence is needed to reflect the entire mental health clinical workforce.

It is therefore good that this study recruited registered clinical staff from a broader range of professional groups: nursing staff, psychiatrists, occupational therapists and psychologists. However limiting recruitment to these four groups means that social workers and members of new and novel clinical groups (such as multi professional approved clinicians) have not been included. This is important given the focus on further developing and expanding these new and novel clinical roles in the NHS long term workforce plan. When discussing recruitment issues with these groups in the past it seems that they face unique retention challenges given the new and novel natures of these roles. It might be worth discussing this in the paper limitations?

I would have appreciated some further detail of who is included in the 'senior managers' recruitment group. For example does this include members of the trust board or finance directors? In the paper it says that this included clinical directors and workforce leads. Does this include HR staff?

Reporting on patient and public involvement

There was very little information about the role of patient and public involvement in the development of this study in the methodology beyond stating: “Patient and public panels were involved in the design and conduct of this study”.

It does however say later in the discussion that “mental health service user groups were consulted during the development of this research project, particularly the formulation of the IPT”. On reviewing the IPT paper (Long et al) I can see that 8 members of a local service user group were involved in the early stages of the IPT development. Perhaps referencing the Long et al paper within the methodology of this paper might be enough to help the reader understand the extent of patient and public involvement in preparing for this study?

Reporting on site selection

Thank you for setting out the criteria for selecting NHS sites. These are clear and seem appropriate.

I appreciate that these sites need to remain anonymous. I would have liked to know more about the demographics of each however I appreciate that this might not be possible based on the details of your ethics application.

VERSION 1 - AUTHOR RESPONSE

Reviewer 1

1. Line 124: the Pawson and Tilly citation needs to be corrected to be in line with the numbered citation system.

Numbered citation added.

4. Throughout the manuscript "trust" is used to describe the organisations. This can lead to challenges in readability at times. I would suggest capitalising as "Trust" to improve readability.

All instances have been changed to Trust as suggested.

Commented [RJ1]: We may need to consider this for the New Roles papers as well.

5. Line 405-406: "where staff reported how senior staff listened but did not respond and feedback" - I think that there is a word missing here... "where staff reported how senior staff listened but did not respond to feedback".

The original sentence is correct but not well articulated. Staff were talking about wanting a ‘you said, we did’ type response where senior staff not only listened but acted and then feedback to staff what had been done. So “respond and feedback” is correct but we have clarified the wording to make this clearer.

6. Line 513-515: "Compulsory leadership and management training for all staff in any leadership or management role will help to improve management in services and make it more supportive and compassionate (39)." - I think that this statement is a bit too definitive. While management training may be helpful, I don't think the evidence supports that it will always create more supportive and compassionate workplaces. I would suggest revising this to be a more speculative suggestion.

We have removed the final part of that sentence and added a new sentence. "Compassionate leadership training can reduce stress and improve staff engagement." P22

7. Line 612 - this statement refers to "survey respondents" which doesn't seem to be aligned to the participants in this study.

It is an error, it should say interview participants, this has been corrected.

Reviewer 2

8. Choice of population and reporting:
I note in the introduction that this paper critiques existing research for only focussing on MH nurses, primary care based workers and psychiatrists when considering the factors that impact retention. The authors suggest that more evidence is needed to reflect the entire mental health clinical workforce.
It is therefore good that this study recruited registered clinical staff from a broader range of professional groups: nursing staff, psychiatrists, occupational therapists and psychologists. However limiting recruitment to these four groups means that social workers and members of new and novel clinical groups (such as multi professional approved clinicians) have not been included. This is important given the focus on further developing and expanding these new and novel clinical roles in the NHS long term workforce plan. When discussing recruitment issues with these groups in the past it seems that they face unique retention challenges given the new and novel natures of these roles. It might be worth discussing this in the paper limitations?

We included all registered professionals who were employed by the organisation. This would have included MPACs (who are usually nurses or psychologists as well), if any of the Trusts had them, which we do not believe they did at the time of our study.

Social workers were included IF they worked for the NHS Trust. However, they are often employed by the local authority rather than the NHS, this would have meant that they were excluded in this case. We have added a passage to the limitations section to make this clear

"As we only included registered staff employed by the NHS organisation we did miss the view points of non-registered staff such as healthcare assistants and registered staff like social workers who are employed by the local authority rather than the NHS." P24

We have also added a line to the sample and recruitment section in the methods to aid clarity. "All registered staff who worked for the organisation were eligible regardless of job title." P8

I would have appreciated some further detail of who is included in the 'senior managers' recruitment group. For example does this include members of the trust board or finance directors? In the paper it says that this included clinical directors and workforce leads. Does this include HR staff?

The different sites provided different people. However, overall it did include board members such as COO, Chief strategy officer, Chief nursing officer, chief medical officer, and Chief People officer.

HR – we had a CPO, 2 heads of HR and 1 deputy CPO.

We also had other senior roles such as head of workforce and head of inclusion. We did not have anyone from finance.

We have added to this part of the results to make it clearer who we spoke to.

"(such as Chief operating officer, chief strategy officer, chief nursing officer, chief medical officer, chief people officer, 2 heads of HR and deputies, EDI leads and workforce leads)," p11

9. Reporting on patient and public involvement

There was very little information about the role of patient and public involvement in the

development of this study in the methodology beyond stating: “Patient and public panels were involved in the design and conduct of this study”.

It does however say later in the discussion that “mental health service user groups were consulted during the development of this research project, particularly the formulation of the IPT”. On reviewing the IPT paper (Long et al) I can see that 8 members of a local service user group were involved in the early stages of the IPT development. Perhaps referencing the Long et al paper within the methodology of this paper might be enough to help the reader understand the extent of patient and public involvement in preparing for this study?

This reference has been added to page 11

10. Reporting on site selection

Thank you for setting out the criteria for selecting NHS sites. These are clear and seem appropriate.

I appreciate that these sites need to remain anonymous. I would have liked to know more about the demographics of each however I appreciate that this might not be possible based on the details of your ethics application.

We can share some limited details, being careful not to identify the sites. So we have added a paragraph to page 8.

Three Trusts were in the South of England, two in the North and 1 in the Midlands. Two served urban populations, the rest served a mixture of rural and urban. Stability index in the year prior to data collection ranged from 0.795 to 0.885. Staff satisfaction ranged from two in the bottom 5 ratings for all English Trusts to two in the top 10 for staff satisfaction.
